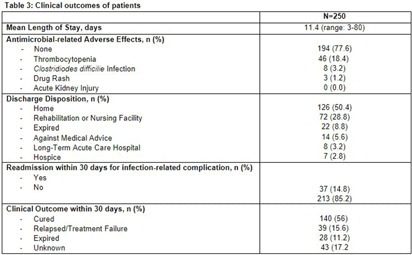# Characteristics, Treatment, and Outcomes of Invasive Group A Streptococcal Infections

**DOI:** 10.1017/ash.2024.176

**Published:** 2024-09-16

**Authors:** Megha Jagannathan, Tamara Jordan, Daniel Kinsey, Rachel Kenney, Michael Veve, Geehan Suleyman, Anita Shallal

**Affiliations:** Henry Ford Hospital

## Abstract

**Background:** Group A Streptococcus (GAS; Streptococcus pyogenes) is an important human pathogen that can cause life-threatening invasive disease, ranging from skin/soft tissue infections to infective endocarditis. In the fall of 2022, the Center for Disease Control & Prevention (CDC) issued an alert due to a global increase in invasive GAS infections, particularly among children and adults with co-morbidities. An increase in invasive disease was observed at our five-hospital healthcare system in Southeast Michigan. The objective of this study was to describe characteristics of patients with invasive GAS and characterize treatment and outcomes of disease. **Methods:** This was a retrospective cross-sectional study of patients from June 2013 to August 2023 with positive blood cultures for GAS. Patients were identified using a data query for positive blood cultures for GAS through Microsoft SQL Server. Patients with age < 1 8 years, polymicrobial bacteremia, incomplete data, or who were enrolled in hospice and/or died within 48-hours of admission were excluded. Collected variables included: demographics, infection characteristics (syndrome, duration of bacteremia), microbiological characteristics (antimicrobial susceptibility testing; AST), antimicrobial treatment (empiric and final, antitoxin therapy), and clinical outcomes (length of hospital stay [LOS], treatment-associated adverse events, 30-day mortality and infection-related readmission). **Results:** 250 patients were included (Table 1). More than half were male with median age of 57.5 years. Diabetes mellitus (38%) and chronic kidney disease (23%) were common comorbidities [Table 1]. Persons experiencing homelessness and persons who use injection drugs accounted for 9% and 13% of the cases, respectively. The most common infective syndrome accompanying bacteremia was cellulitis (57%). The majority of patients received vancomycin for empiric therapy (81%) and penicillin (38%) or cephalosporin (36%) for final regimen [Table 2]. A total of 79 GAS isolates (32%) were clindamycin resistant. Clindamycin was included in the empiric regimen of 20 (8%) patients, the final regimen in 44 (18%) of patients, and as antitoxin adjunct therapy in 135 (54%) of patients. A third (33%) of patients received no antitoxin. The average duration of antitoxin therapy was 3.6 days and antimicrobial therapy 19.9 days. The mean LOS was 11.4 days (Table 3). Thirty nine (16%) patients had treatment failure and 8 (3%) experienced C. difficile infection within 30 days of antimicrobial treatment. Thirty-day mortality was 11%; of these, 9% had in-hospital mortality. **Conclusions:** Invasive GAS infection confers significant morbidity and mortality, and ongoing research is needed to determine the best treatment regimens in the era of increasing clindamycin resistance.

**Figure:**
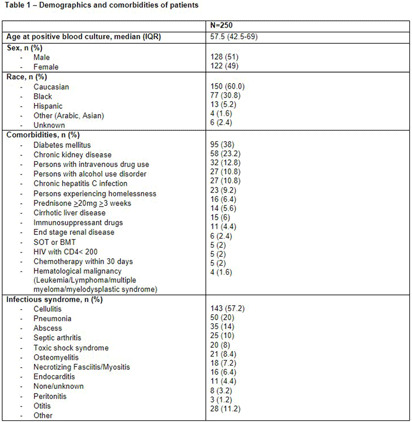

**Figure:**
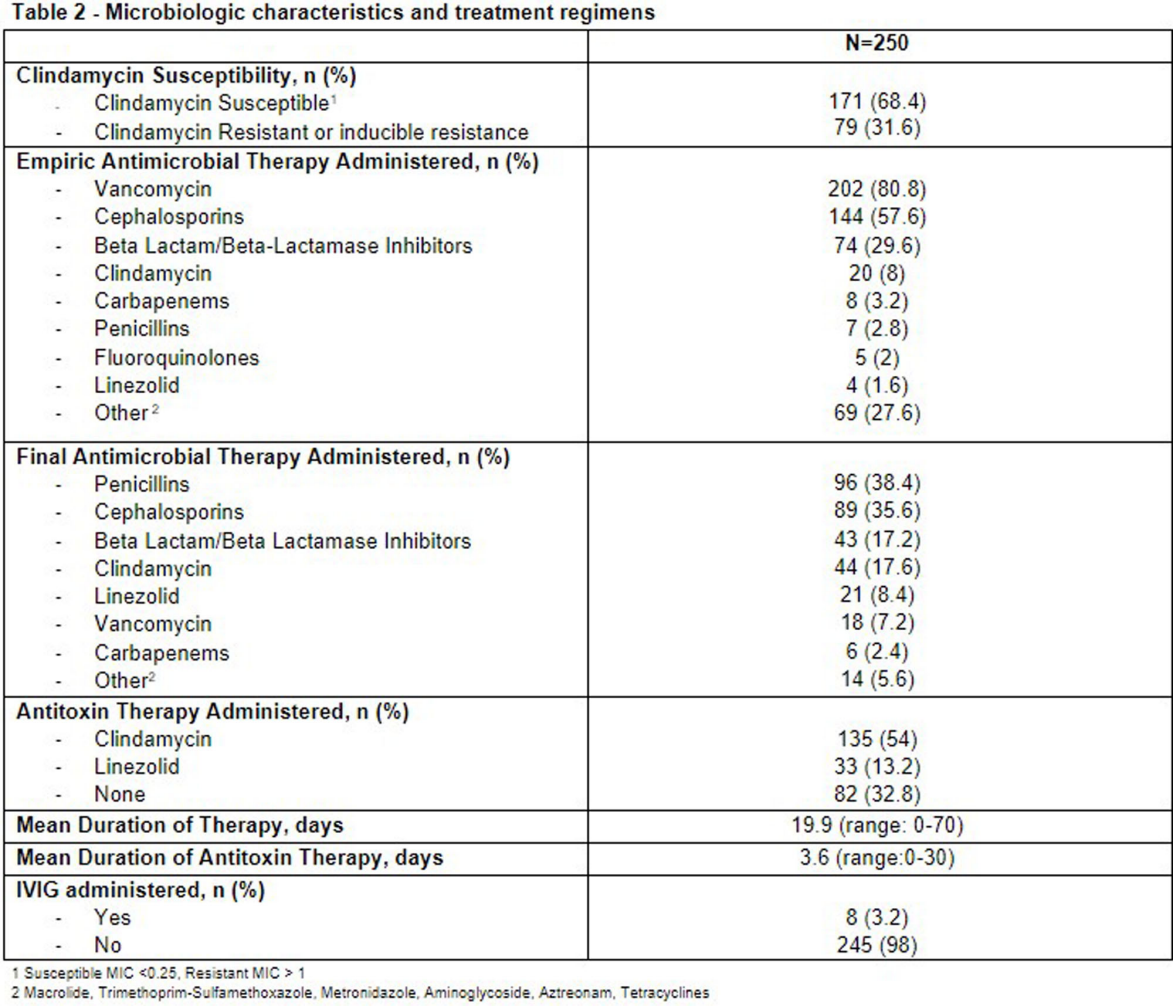

**Figure:**